# Gut microbiota in reintroduction of giant panda

**DOI:** 10.1002/ece3.5963

**Published:** 2020-01-06

**Authors:** Jingsi Tang, Chengdong Wang, Hemin Zhang, Jiangchao Zhao, Wei Guo, Sudhanshu Mishra, Fanli Kong, Bo Zeng, Ruihong Ning, Desheng Li, Jiandong Yang, Mingyao Yang, Mingwang Zhang, Qingyong Ni, Yan Li, Ying Li

**Affiliations:** ^1^ Farm Animal Genetic Resources Exploration and Innovation Key Laboratory of Sichuan Province Sichuan Agricultural University Chengdu Sichuan China; ^2^ China Conservation and Research Center for the Giant Panda Ya'an Sichuan China; ^3^ Department of Animal Science Division of Agriculture University of Arkansas Fayetteville AR USA

**Keywords:** *Ailuropoda melanoleuca*, giant panda, gut microbiota, reintroduction, wild‐training

## Abstract

Reintroduction is a key approach in the conservation of endangered species. In recent decades, many reintroduction projects have been conducted for conservation purposes, but the rate of success has been low. Given the important role of gut microbiota in health and diseases, we questioned whether gut microbiota would play a crucial role in giant panda's wild‐training process. The wild procedure is when captive‐born babies live with their mothers in a wilderness enclosure and learn wilderness survival skills from their mothers. During the wild‐training process, the baby pandas undergo wilderness survival tests and regular physical examinations. Based on their performance through these tests, the top subjects (age 2–3 years old) are released into the wild while the others are translocated to captivity. After release, we tracked one released panda (Zhangxiang) and collected its fecal samples for 5 months (January 16, 2013 to March 29 2014). Here, we analyzed the Illumina HiSeq sequencing data (V4 region of 16S rRNA gene) from captive pandas (*n* = 24), wild‐training baby pandas (*n* = 8) of which 6 were released and 2 were unreleased, wild‐training mother pandas (*n* = 8), one released panda (Zhangxiang), and wild giant pandas (*n* = 18). Our results showed that the gut microbiota of wild‐training pandas is significantly different from that of wild pandas but similar to that of captive ones. The gut microbiota of the released panda Zhangxiang gradually changed to become similar to those of wild pandas after release. In addition, we identified several bacteria that were enriched in the released baby pandas before release, compared with the unreleased baby pandas. These bacteria include several known gut‐health related beneficial taxa such as *Roseburia*, *Coprococcus*, *Sutterella, Dorea,* and *Ruminococcus*. Therefore, our results suggest that certain members of the gut microbiota may be important in panda reintroduction.

## Graphical abstract

The gut microbiota of wild‐training pandas is significantly different from that of wild pandas, but similar to that of captive ones. However, the gut microbiota of panda Zhangxiang gradually changed to that of wild pandas after successful release. Several bacteria that were enriched in the wild‐training baby pandas who met the criteria for release into the wild. These bacteria include several known gut‐health‐related beneficial taxa such as Roseburia, Coprococcus, Sutterella, Dorea, and Ruminococcus, indicating potential roles of these bacteria in the fitness of these baby pandas.

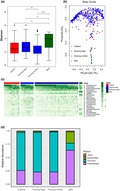

## 1 INTRODUCTION

Conservation translocation is the deliberate movement of a species from one site to another to save endangered species from extinction (Germano et al., 2015). According to the International Union for Conservation Union (IUCN), translocation includes introduction, reintroduction, and restocking (IUCN, 2013) of endangered species. Of these, reintroduction is the most common strategy. Translocation moves a species from captivity or other areas where the organism survives, into another area within their original geographic range. This usually occurs where populations have significantly declined or disappeared due to natural catastrophes or human interference (Yang et al., 2018). Reintroduction refers to the intentional movement of captive‐born organisms into, or near, the species’ natural historic range to reestablish or augment a wild population (Beck, Rapaport, Price, & Wilson, 1994). Many reintroduction programs involving endangered or vulnerable species have been carried out for conservation purposes worldwide, such as that of black bears, *Ursus americanu*, (Clark, Huber, & Servheen, 2002), Mexican wolves, *Canis lupus baileyi*, (Oakleaf, Stark, Overy, & Smith, 2004), and giant pandas, *Ailuropoda melanoleuca*, (Shan et al., 2014; Yang et al., 2018). However, the average success rate of reintroduction is estimated to be between 26% and 32% from 2002 to 2014 (Fischer & Lindenmayer, 2000; Jule, Leaver, & Lea, 2008), which suggests that the technique of reintroductions needs further investigation and improvement in order to ensure that they are viable options (Fischer & Lindenmayer, 2000; Seddon, Strauss, & Innes, 2012). To improve the success of reintroduction, a series of standards for documenting and monitoring the methods and outcomes of such a practice is essential (Sutherland et al., 2010).

Through decades of conservation efforts, the giant panda was upgraded from an endangered species to the vulnerable category in 2016. This was an important change; however, it is vital that such efforts continue so as to reinforce this species’ survival (Swaisgood, Wang, & Wei, 2017). Although both the number of protected areas for pandas and the number of captive‐born and wild pandas have increased in recent decades, the wild panda population has, presently, only 33 isolated subpopulations and of these, only 6 have more than 100 pandas. This low population level is due to such adverse factors such as roads, hydroelectric dams, mining, tourism (Administration, 2015), and climate change, which continue to fragment and degrade panda habitats. For example, genetic research of pandas in the Xiaoxiangling Mountains estimated that the population has a very high risk of extinction if it remains isolated with a low gene flow (Zhu, Zhan, Meng, Zhang, & Wei, 2010; Zhu, Zhang, Gu, & Wei, 2011; L. F. Zhu, Zhan, Wu, et al., 2010). For fragmented or isolated habitat patches, habitat corridors have been planned or constructed to facilitate dispersal and gene flow. For small and isolated populations, translocation or reintroduction programs have been implemented to improve reproduction success and genetic diversity. Conservationists adopted a translocation and reintroduction pilot plan for genetic rescue involving the release of 3 rescued wild‐caught pandas and 9 captive‐born pandas into the Xiaoxiangling Mountains. Before captive‐born pandas released into the wild, they have to go through the wild training. The wild‐training process is when captive‐born baby pandas live with their mothers and learn survival skills from them in a natural fence with limited human interference. The top performers were released into the wild and then monitored using GPS. Unfortunately, 3 of the 9 released captive‐born pandas died between 2006 and 2017. One death was the result of fighting with wild pandas, and the cause of the other 2 remains unknown. Due to this, the efficiency of the wild‐training method for captive pandas has come into question.

Gut microbiota of mammals has emerged as an important factor in maintaining host health and well‐being (Clemente, Ursell, Parfrey, & Knight, 2012; Long, Gahan, & Joyce, 2017; Quigley, 2013; Rooks & Garrett, 2016). For the giant panda, gastrointestinal diseases are the most common causes of mortality in both captive and wild pandas (Janssen et al., 2006), which suggests that gut microbiota may play an important role in giant panda's health. In addition, several studies have found that certain members of the gut microbiota of pandas play a leading role in the digestion of their unique bamboo diet (Wei, Wang, & Wu, 2015; Zhang et al., 2018; Zhu, Wu, Dai, Zhang, & Wei, 2011). Multiple factors also had an effect on giant panda's gut microbiota, such as seasonal variations (Xue et al., 2015), age (Zhang et al., 2018) and captivity (Wei et al., 2015). Despite sharing the same diet, the gut microbiota of the giant panda is distinct from that of the red panda and is clustered more closely to that of the black bear (Y. Li, Guo, et al., 2015). This implies that the evolution of the gut microbiota of pandas is based more on host phylogeny than a diet. Thus, the gut microbiota is important for giant panda's health and survival. Several studies have reported monitoring data of the wild‐training process for panda reintroduction, including activity patterns, genetic analysis, and foraging strategies (Lei et al., 2015; Yang et al., 2018). What remains unclear is, to what extent does the wild‐training process of captive‐born baby pandas influence their gut microbiota? In this study, we sequenced the V4 region of 16S rRNA gene of 463 fecal samples from pandas which were captive, wild training, wild, and released, the released panda being (Zhangxiang). Our findings characterized the gut microbiota of captive‐born baby pandas longitudinally during the wild‐training process and found that the gut microbiota communities of these baby pandas were similar to those of captive‐born pandas. Interestingly, the gut microbiota of baby panda Zhangxiang gradually developed into a stage similar to those of wild pandas after being released into the wild.

## 2 MATERIALS AND METHOD

### 2.1 Ethics statement

All animal work was carried out under the approval of the Institutional Animal Care and Use Committee of the Sichuan Agricultural University under the permit number DKYB20150301. All experiments were performed in accordance with the approved guidelines and regulations.

### 2.2 Sample collection

**Figure 1:**
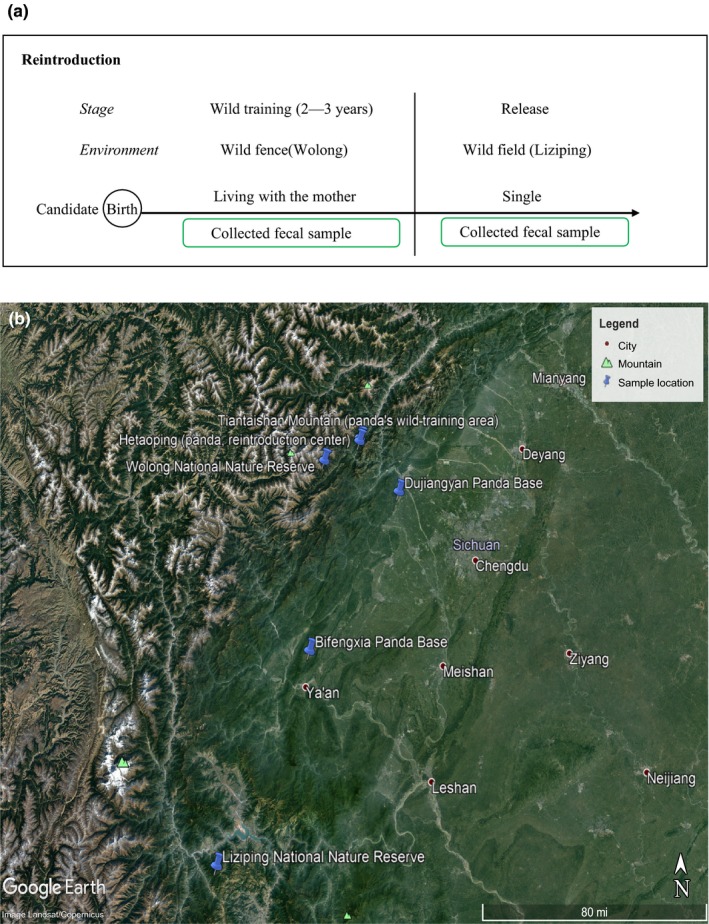
Reintroduction process and sample location in this study. (a) Reintroduction process. (b) Sample location of giant panda in this study

A total of 463 fresh fecal samples from giant pandas were collected from 2012 to 2015. Fresh feces were frozen upon collection and shipped on dry ice to our laboratory for analysis. Each sample was assigned to 1 of 4 groups: captive, wild training, wild, and one released panda (Zhangxiang). Eighty‐seven fecal samples were collected from 24 captive pandas living in the giant panda base (Dujiangyan base/Bifengxia base/Hetaoping base, see Figure 1b) based on defecation observation (captive group). Three hundred and fifteen fecal samples were collected from 16 wild‐training pandas (baby: *n* = 8, sample size = 74 mother: *n* = 8, sample size = 241) living in wild‐training areas based on defecation observation. Eighteen fresh samples with mucosa were collected from 18 pandas which lived in the Wolong National Nature Reserve (wild group) based on freshness level. Individual genotypes were identified by Qiao et al (Qiao et al., 2019). The identification information is provided in Table 1. Forty‐three fresh fecal samples were collected from panda Zhangxiang (ZX Released group) who was released into Liziping National Nature Reserve in the Xiaoxiangling Mountains on November 6, 2013. We positioned ZX using GPS and collected fecal samples weekly from November 6, 2013, to April 27, 2014. Sample metadata information was recorded in Table S1.

**Table 1:** Identification information of wild giant panda

Qiao's ID	Sample ID	Sequencing ID	Sample time	Longitude	Latitude	Individual identification
63	W2	D160110104	2015.3.27	30.9746	103.1650	YES
64	W3	D160110105	2015.3.28	30.9950	103.1808	YES
66	W4	D160110106	2015.3.29	30.9724	103.1522	YES
86	W6	D16031511	2015.3.29	31.0660	103.2455	YES
95	W8	D160110110	2015.4.2	31.0899	103.2447	YES
99	W10	D160110112	2015.4.3	31.0075	103.1751	YES
100	W11	D160110113	2015.4.4	30.9239	103.2692	YES
107	W14	D160110115	2015.4.20	31.1542	103.2803	YES
110	W17	D160110118	2015.4.20	31.1617	103.3367	YES
113	W20	D160110121	2015.4.23	31.1047	103.3429	YES
114	W21	D160110122	2015.4.20	31.1554	103.2812	YES
115	W22	D160110123	2015.4.20	31.1552	103.3485	YES
116	W23	D160110124	2015.3.29	31.0709	103.2480	YES
119	W25	D160110126	2015.3.30	31.0354	103.2688	YES
124	W27	D16031513	2015.3.29	31.0568	103.2552	YES
132	W28	D160110130	2015.3.29	30.9435	103.2292	YES
133	W29	D160110131	2015.3.29	30.9747	103.3250	YES
134	W30	D160110132	2015.3.29	30.9535	103.2384	YES

### 2.3 Wild‐training process

In this study, we collected opportunistically a total of 74 fecal samples from eight baby pandas undergoing the wild‐training process. These samples are the subset of the wild‐training group, and we grouped them into released (*n* = 6 with 38 fecal samples from 2012 to 2015) group and unreleased (*n* = 2 with 36 fecal samples from 2013 to 2015) group. According to the reintroduction process (Figure 1a), the wildness procedure started once the baby pandas are born. They live with their mothers in the wild enclosure and learn wilderness survival skills from their mothers. During the wild‐training process, the baby pandas are scored by their regular physical examinations, and their capabilities to identify and respond to natural enemies, recognize the same species, and choose a safe resting place. Based on their performance through these tests, the top subjects (age 2–3 years old) are released into the wild and others are translocated to captivity. We defined the babies which had been released after training as the released group and defined babies which had been returned to captivity after training as the unreleased group. The wild‐training area (Hetaoping or Tiantaishan in Figure 1b) is located in Sichuan Wolong Nature Reserve which is a type of deciduous broad‐leaved forest, and the area is about 100–120 hm^2^. The altitude is about 1860–3010 m and bamboos, a giant panda staple food, are present.

### 2.4 DNA extraction, amplicon PCR, and sequencing

Total DNA was extracted from each sample using the UPure Stool DNA Kit (Biobase Technologies Co., Ltd) according to the manufacturer's protocol. The quality of DNA was measured by using a NanoDrop Spectrophotometer (Thermo Fisher Scientific, Inc.), agarose gel electrophoresis, and Qubit 2.0 (Thermo Fisher, Inc.). Only DNA samples that met these criteria (DNA concentration ≥5 ng/µL, OD_260/280_ = 1.8 and total volume ≥150 ng) were used for further analysis. Bacterial 16S rRNA gene amplicons were produced and sequenced at the Novogene Bioinformatics Technology Co., Ltd. Variable region 4 of the 16S rRNA gene was amplified using the 515f/806r barcoded primer pair (515f: 5'‐GTGCCAGCMGCCGCGGTAA‐3', 806r: 5'‐GGACTACHVGGGTWTCTAAT‐3') (Caporaso et al., 2011). PCRs were performed in triplicate and amplicons from the same sample were mixed and purified. High‐throughput sequencing was conducted to obtain paired‐end 250 bp sequences by using the Illumina Hiseq 2,500 platform (Illumina). Negative controls (no sample added) were included in both the DNA extraction and PCR amplification protocols to test for the presence of contamination. The negative controls yielded negligible DNA concentrations thus indicating the absence of contamination.

### 2.5 Sequence processing and analysis

Data analysis was performed using QIIME2 (version: 2019.1) pipeline with default parameters. Raw sequences were demultiplexed using the script of Novogene to generate per sample FASTQ sequence files. To obtain a high‐resolution analogue of amplicon sequence variant (ASV) table, DADA2 (Callahan et al., 2016) was used for detecting and correcting Illumina amplicon sequence errors. Sequence data were denoised, dereplicated, chimera removed, and merged of paired‐end reads with 200‐bases for each forward and reverse read using the DADA2 (denoised‐paired with default parameters). Subsequently, the taxonomy assignment was performed using the scikit‐learn method (version: 0.19.1) against the Greengenes database (gg_13_8) with a 99% similarity threshold. Mitochondria and chloroplast sequences were removed using filter‐seqs and filter‐table command. Taxonomy classification was performed according to the QIIME2 workflow and the relative abundance was shown by barplot using R package. To correct for differences in sequencing depth, we randomly subsampled the sequences from each sample (2,383 sequences per sample) for computing the alpha diversity metrics (Shannon index; Shannon, 1948), Observed OTUs, Evenness and Faith's Phylogenetic Diversity (Faith, 1992) and beta diversity metrics (Jaccard distance, Bray–Curtis distance, unweighted UniFrac distance and weighted UniFrac distance). Principal coordinate analysis (PCoA) and a Heatmap were produced by version 3.4.3 of R (Team, 2017).

### 2.6 Random forest classification

Random Forest was used to identify microbial signatures that best differentiated between released and unreleased groups from the training baby pandas before release. Random Forest is a robust machine‐learning technique that accounts for the nonlinear relationships and dependencies among microbiota features. Alpha diversity measures and relative abundance of the top 100 OTUs that accounts for 98.99% of the sequences were used as inputs (predictors) for the model. A variable importance plot was generated by ranking the variables with their importance scores (mean decrease accuracy or MDA). The top variable in the plot is defined as the most predictive. A supervised Random Forest was performed by using AUCRF package in R version 3.4.3 with 10,000 trees. We used the default setting for “mtry”, which is the square root of the number of variables.

### 2.7 Statistical analysis

The Mann–Whitney *U* test and the Kruskal–Wallis test were used to determine significant differences among the captive, training baby, training mother, and wild groups using alpha diversity measures. Permutational multivariate analysis of variance (PERMANOVA) test was used to determine the strength and significance of given factors (Lifestyle/Season/Age, strata = Individual) in explaining microbiota variation between comparison groups (Table 2). Analysis of similarities (ANOSIM) was used to evaluate the similarity between groups (Table 3). LDA Effect Size (LEfSe) (Segata et al., 2011) was used to identity bacteria with significant differences in abundance between groups. Area under the curve (AUC) was used to measure the predictive accuracy of Random Forest). All the statistical analysis run in R version 3.4.3. Significance was set at *p* < .05.

**Table 2:** PERMANOVA for specific factors (lifestyle/season/age/individual)

PERMANOVA results for lifestyle/season (strata = Individual)							
Sample size = 420	Df	Sums of squares	Mean squares	F.Model	Variation (R2)	Pr (>F)	Signif
Lifestyle (captive/training/wild)	2	6.441	3.2205	20.5699	0.08261	0.001	***
Season (spring/summer/autumn/winter)	3	6.191	2.0638	13.1818	0.0794	0.001	***
Lifestyle:season	3	0.993	0.331	2.1142	0.01274	0.449	
Residuals	411	64.347	0.1566		0.82525		
Total	419	77.972			1		

PERMANOVA results for lifestyle/season/age (strata = Individual)							
Sample size = 402	Df	Sums of squares	Mean squares	F.Model	Variation (R2)	Pr (>F)	Signif
Lifestyle (captive/training)	1	0.237	0.237	1.611	0.003	0.355	
Season (spring/summer/autumn/winter)	3	6.191	2.064	14.024	0.091	0.001	***
Age (subadult/adult)	1	0.845	0.845	5.743	0.012	0.001	***
Lifestyle:season	3	1.011	0.337	2.289	0.015	0.308	
Lifestyle:age	1	0.750	0.750	5.097	0.011	0.024	*
Season:age	3	1.761	0.587	3.988	0.026	0.001	***
Lifestyle:season:age	2	0.636	0.318	2.162	0.009	0.145	
Residuals	387	56.953	0.147		0.833		
Total	401	68.384			1.000		

PERMANOVA results for lifestyle/age (strata = Individual) on spring							
Sample size = 124	Df	Sums of squares	Mean squares	F.Model	Variation (R2)	Pr (>F)	Signif
Lifestyle (captive/training)	1	0.1932	0.193203	1.46194	0.011035	0.811	
Age (adult/subadult)	1	1.35213	1.352131	10.2313	0.077232	0.811	
Lifestyle:age	1	0.10347	0.103474	0.78297	0.00591	0.666	
Residuals	120	15.8587	0.132156		0.905823		
Total	123	17.5075			1		

PERMANOVA results for lifestyle/age (strata = Individual) on winter							
Sample size = 123	Df	Sums of squares	Mean squares	F.Model	Variation (R2)	Pr (>F)	Signif
Lifestyle (captive/training)	1	0.41391	0.413905	2.99859	0.024138	0.114	
Age (adult/subadult)	1	0.03797	0.037969	0.27507	0.002214	0.94	
Lifestyle:age	1	0.26964	0.269643	1.95346	0.015725	0.839	
Residuals	119	16.426	0.138033		0.957923		
Total	122	17.1475			1		

PERMANOVA results for lifestyle/age (strata = Individual) on autumn							
Sample size = 86	Df	Sums of squares	Mean squares	F. Model	Variation (R2)	Pr (>F)	Signif
Lifestyle (captive/training)	1	0.3792	0.379199	2.44742	0.028234	0.03	*
Age (adult/subadult)	1	0.14511	0.14511	0.93657	0.010805	0.072	.
Lifestyle:age	1	0.20115	0.201148	1.29825	0.014977	0.314	
Residuals	82	12.7049	0.154938		0.945984		
Total	85	13.4304			1		

PERMANOVA results for lifestyle (strata = Individual) on summer							
Sample size = 69	Df	Sums of squares	Mean squares	F. Model	Variation (R2)	Pr (>F)	Signif
Lifestyle (captive/training)	1	0.62427	0.624271	3.09483	0.044152	0.142	
Residuals	67	13.5149	0.201714		0.955848		
Total	68	14.1391			1		

Signif. codes: 0 “***” 0.001 “**” 0.01 “*” 0.05 “.” 0.1 “ ” 1.

**Table 3:** ANOSIM analysis for beta diversity

Group 1	Group 2	Sample size	Permutations	R	*p*‐value	q‐value
ANOSIM based on Bray–Curtis distance among captive, training baby, training mother, and wild group						
Captive	Training_baby	161	999	0.014142	.091	0.1092
Captive	Training_mother	328	999	0.040726	.063	0.0945
Captive	Wild	105	999	0.732841	.001	0.002
Training_baby	Training_mother	315	999	0.025264	.182	0.182
Training_baby	Wild	92	999	0.819341	.001	0.002
Training_mother	Wild	259	999	0.813421	.001	0.002
ANOSIM for unweighted UniFrac distance between released and unreleased group						
Released	Unreleased	74	999	0.190186	.001	0.001

## 3 RESULTS

### 3.1 The gut microbiota of wild‐training baby pandas is more similar to the captive pandas

**Figure 2:**
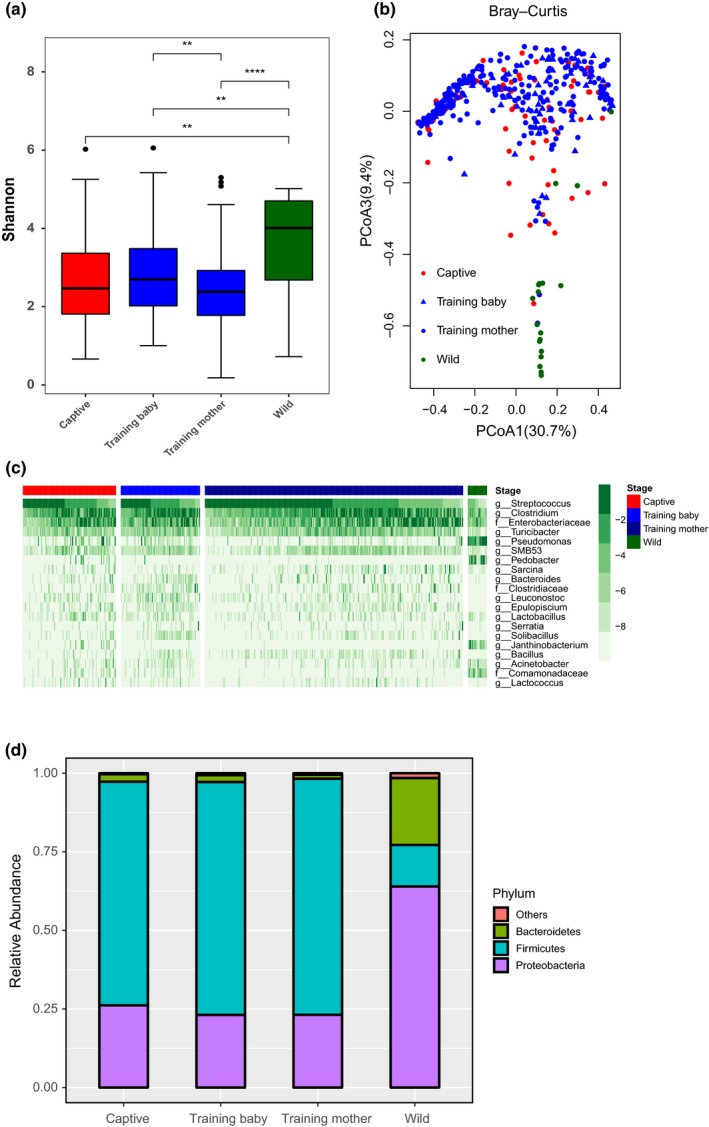
Gut microbiota of wild‐training baby pandas varies from that of wild pandas. (a) Shannon diversity among captive, training baby, training mother, and wild pandas. The Kruskal–Wallis test was used for the comparison. * stands for *p* < .05, ** stands for *p* < .005, and **** stands for *p* < .00005. (b) PCoA plot based on Bray–Curtis among captive, training baby, training mother, and wild pandas. (c) Heatmap of top 20 bacteria in genera level among captive, training baby, training mother and wild pandas. (d) Relative abundance in phylum level among captive, training baby, training mother, and wild pandas

**Figure 3:**
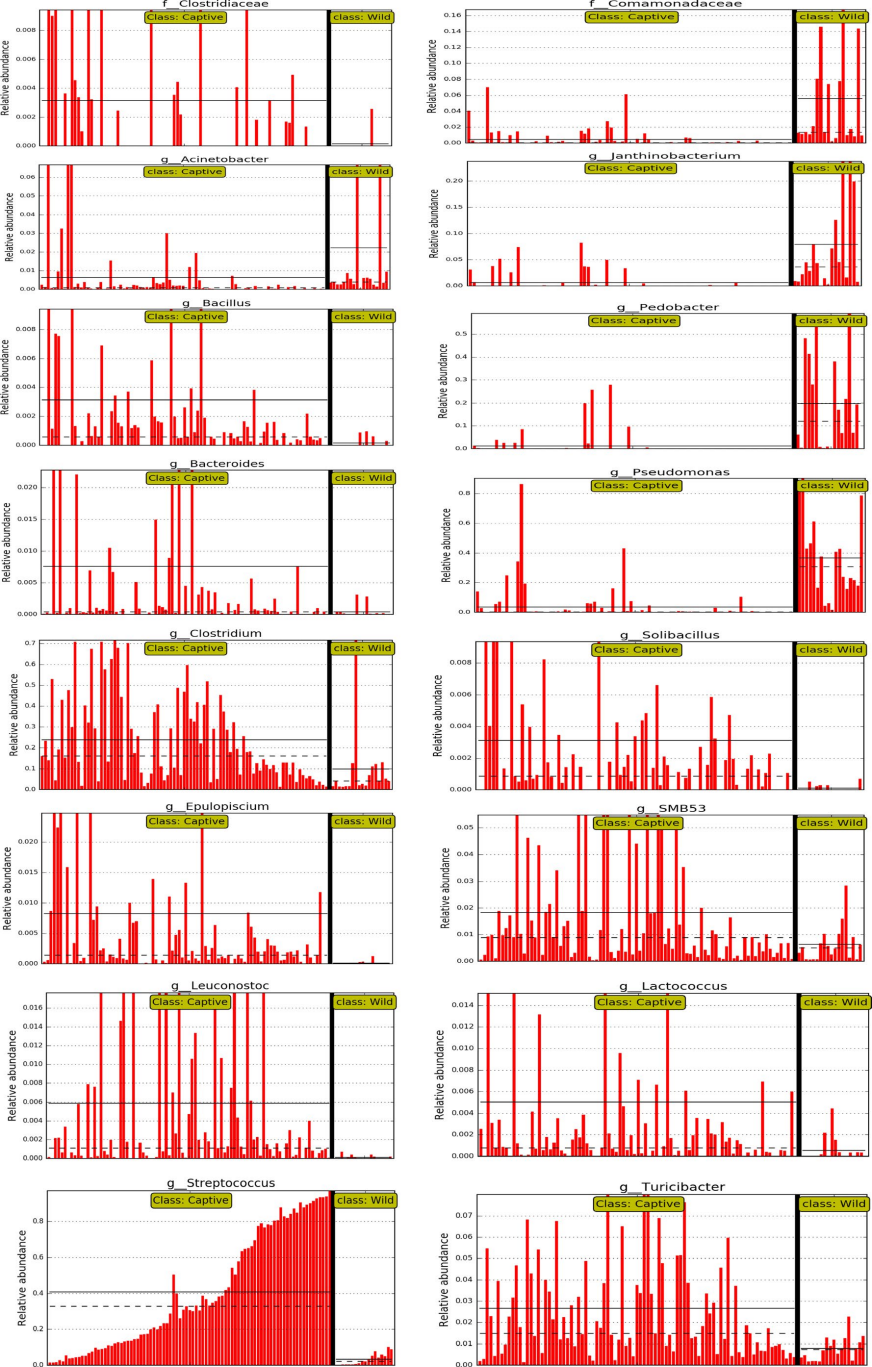
Different gut microbiota in captive groups compared with wild group based on LEfSe analysis

**Figure 4:**
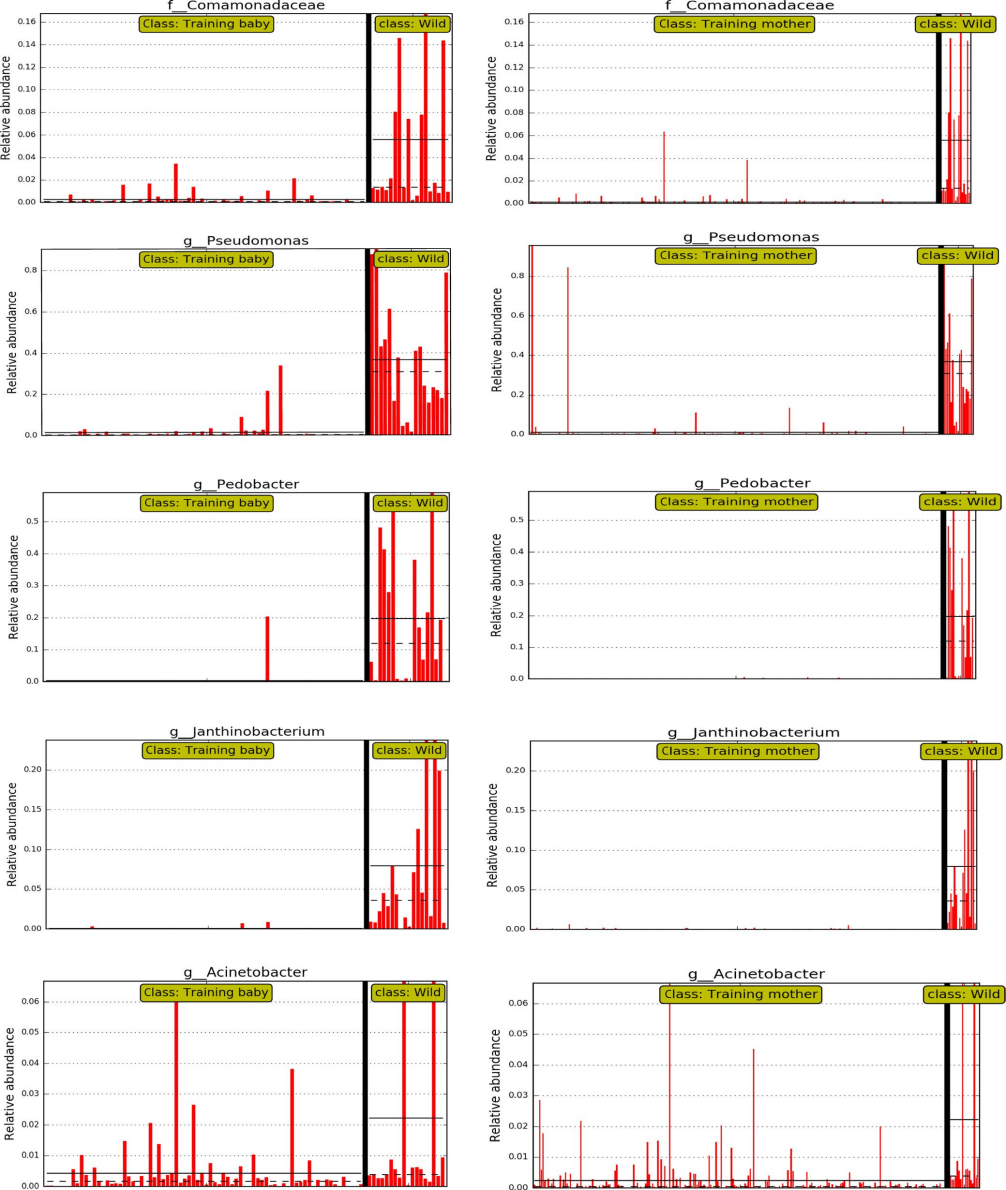
Different gut microbiota in training baby and training mother groups compared with wild group based on LEfSe analysis

We collected fresh fecal samples from baby pandas and their mothers in areas of wild training, as well as from captive and wild pandas. We first calculated the Shannon index to assess the within‐sample diversity among captive, training baby, training mother, and wild groups. As shown in Figure 2a, the alpha diversity of the gut microbiota in training baby, training mother, and captive group were significantly lower than that of the wild group using the Mann–Whitney *U* test. Also observed, the diversity of training babies was significantly higher than that of the mothers. The between‐group dissimilarity (i.e., beta diversity) was assessed using Bray–Curtis distance visualized by PCoA (Figure 2b). The gut microbiotas in the wild group were significantly different from those of the training babies (*R*‐value = 0.82, *p*‐value = .001, *q*‐value = 0.002), training mothers (*R*‐value = 0.81, *p*‐value = .001, *q*‐value = 0.002), and captive pandas (*R*‐value = 0.73, *p*‐value = .001, *q*‐value = 0.002) by ANOSIM analysis. In addition, there are several factors influencing the gut microbiota of pandas involved in this study, such as season variation, individual, and lifestyle. Our PERMANOVA results showed that both lifestyle and season were the influential factor that explained variation between samples based on the bacteria abundance which account for 94% of total reads (Table 2). However, when we excluded the wild group and compared these three factors (lifestyle/season/age, strata = individual), the season and age became the influential factors. Subsequently, we compared the two factors (lifestyle/age, strata = individual) in the same season using 402 fecal samples (Table 2). Age is not a significant factor across all comparisons, but the lifestyle factor is significant in autumn. Also observed, the bacterial community composition of the training baby pandas was more similar to the captive ones but different from the wild pandas at both the genera and phylum level (Figures 2d1c). A heatmap shows the different relative abundances of the top 20 genera of these groups which accounted for 94% of the total reads. The bacterial community compositions of the training baby, training mother, and captive groups were clearly different from that of the wild group through the LEfSe analysis (Figures 3, 4). *Streptococcus, Clostridium,* and *Enterobacteriaceae* were the predominant genera in training baby (32 ± 27%, 28 ± 20%, 17 ± 20%), training mother (40 ± 31%, 23 ± 20%, 20 ± 24%), and captive group (39 ± 31%, 22 ± 19%, 18 ± 22%). *Pseudomonas* and *Pedobacter* as the predominant taxon in the wild group (31 ± 26%, 16 ± 17%). Firmicutes were the top abundant phyla in the training baby (74 ± 26%), training mother (75 ± 27%), and captive group (71 ± 27%), whereas Proteobacteria (64 ± 21%) were the top phylum in the wild group. Our results demonstrated that the gut microbiota of the training baby pandas was more similar to their mothers and the captive pandas than the wild pandas.

### 3.2 The gut microbiota of the released panda ZX gradually converged into that of wild pandas

**Figure 5:**
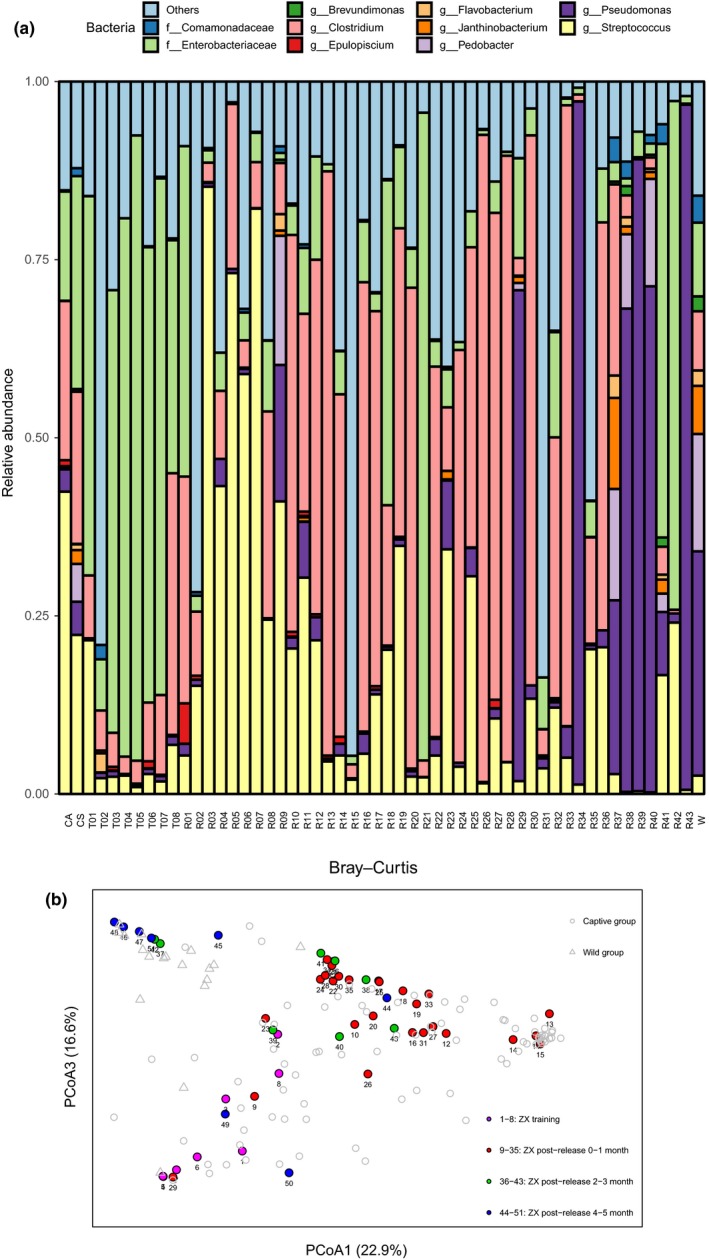
Gut microbiota of the postrelease panda, Zhangxiang, gradually transforms into that of wild pandas. (a) Relative abundance in genus level among captive adult (CA), captive subadult (CS), ZX training (T), ZX released (R), and wild pandas (W). The T and R groups were arranged by the time. (b) PCoA plot based on Bray–Curtis for gut bacteria beta diversity of Zhangxiang panda from prerelease to postrelease. Gray circle stands fecal samples of captive pandas (*n* = 24). Gray triangle stands the fecal samples of wild pandas (*n* = 18). Purple circle stands the fecal samples of ZX panda (1.5–2 years old) in the wild‐training process. Red circle stands the fecal samples of ZX panda (2 years old) after released into the wild in the first month. Green circle stands the fecal samples of ZX panda (2 years old) after released into the wild in the 2–3 month. Blue circle stands the fecal samples of ZX panda (2 years old) after released into the wild in the 4–5 month

The above‐mentioned data show that the gut microbiotas of the training babies were more similar to those of mothers and the captive pandas. We next sought to examine how the gut microbiota changed after a successful release into the wild. We tracked one baby panda (Zhangxiang, ZX) and collected its fecal samples after release. In the Wolong Nature Reserve, ZX mainly ate bamboos and other unknown foods. But in the wild‐training process, ZX mainly ate the bamboos and breastmilk present in the natural enclosure. Figure 5a displays the gradual changing of the bacteria community composition from the training to the postrelease of a baby panda Zhang Xiang (ZX). During this transition, ZX had a gradual reduction in the abundance of *Enterobacteriaceae* and *Streptococcus* and an increase in *Pseudomonas*. Interestingly, both *Enterobacteriaceae* and *Streptococcus* were rich in the captive group, and *Pseudomonas* was abundant in the wild pandas. Moreover, the gut microbiota structure of ZX gradually clustered with that of wild pandas, especially at 4–5 months after release (Figure 5b). These results indicated that the gut microbiota of ZX in the wild‐training group was similar to that of the captive group but gradually evolved into a community characteristic of wild pandas after a successful release.

### 3.3 Important bacterial taxa of baby giant panda during the wild‐training process

**Figure 6:**
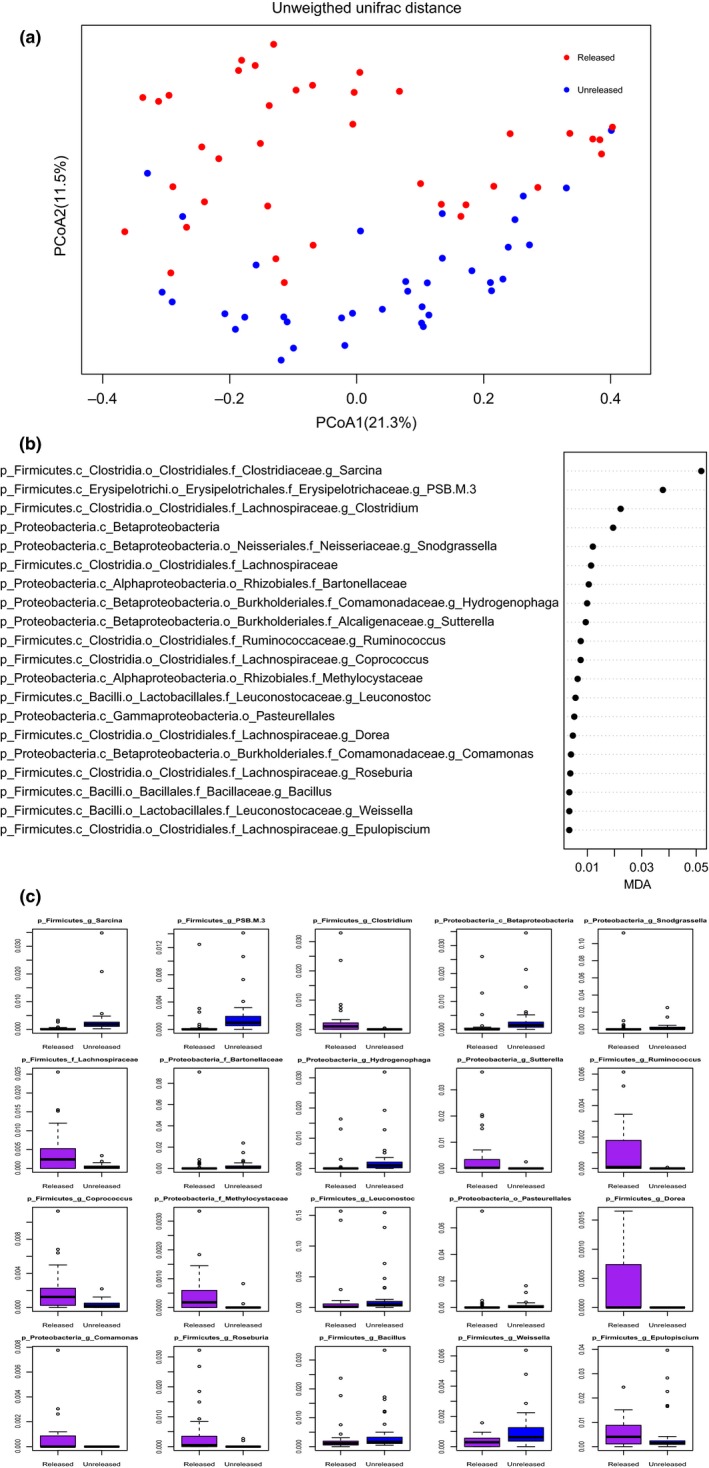
Identification of the important bacterial taxa of baby pandas from the wild‐training group. (a) PCoA plot based on unweighted UniFrac distance for beta diversity between released and unreleased groups. (b) The bacteria of greatest difference between released and unreleased groups using Random Forest. (c) The Relative abundance of bacteria of greatest difference between released and unreleased groups

Finally, we assessed whether the gut microbiota is related with the releasable babies during the wild‐training process. The releasable panda was the top subject based on the performance of survival tests. To this end, we compared the beta diversity of the gut microbiota of the released and unreleased groups. The released group and unreleased group are the subsets of wild‐training pandas group based on whether the babies were released or not after training. As shown in Figure 6a, the bacterial communities of the released group (*n* = 6, fecal sample = 38) were different from those of the unreleased group (*n* = 2, fecal sample = 36) (ANOSIM, *R* = 0.19, *p*‐value = .001, *q*‐value = 0.001). To determine if the gut bacteria of baby pandas may be used as an index of releasable panda selection during the wild training, we used Random Forest to identify microbial signatures that best differentiated between the released and the unreleased groups. In the Random Forest model, each feature was assigned an MDA based on the increase in error caused by removing that feature from the predictor list. The features were ranked by their importance scores which were considered highly predictive. The bacterial taxa identified by random forest accurately predicted the important gut microbiota of releasable baby pandas in the wild‐training process, with an area under the curve (AUC) value of 0.9737 (sensitivity = 0.944, specificity = 0.947). The top 20 microbial signatures that distinguished the released from the unreleased group included 12 Firmicutes and 8 Proteobacterias (Figure 6b). Among the top 20 predictors, seven bacteria were more abundant in the unreleased group (e.g., g_Sarcina, g_PSB_M_3, g_Betaproteobacteria g_Hydrogenophaga, g_Leuconostoc, g_Bacillus, g_Weissella). Also, ten bacteria were enriched in the released group (e.g., g_Clostridium, f_Lachnospiraceae, g_Sutterella, g_Coprococcus, g_Ruminococcus, f_Methylocystaceae, g_Dorea, g_Comamonas, g_Epulopiscium, g_Roseburia) (Figure 6c).

## 4 DISCUSSION

**Figure 7:**
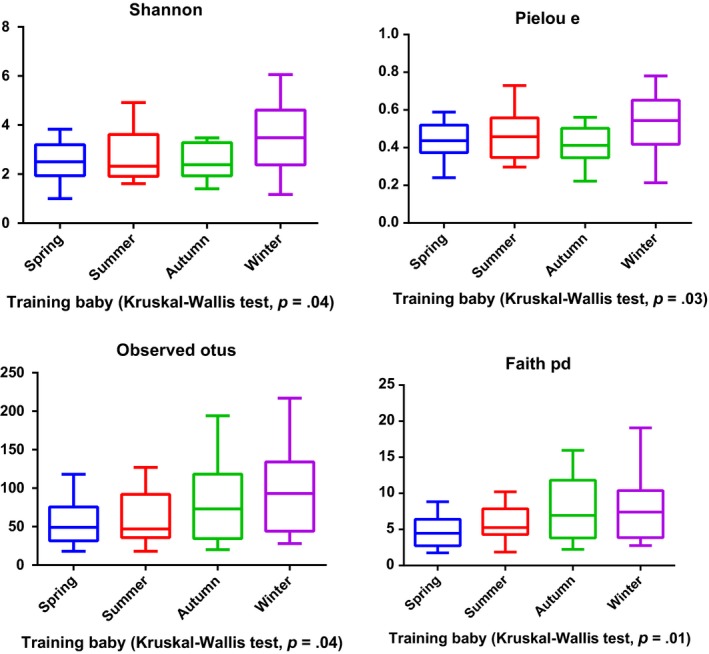
Alpha diversity of gut microbiota in training baby pandas across seasons

**Figure 8:**
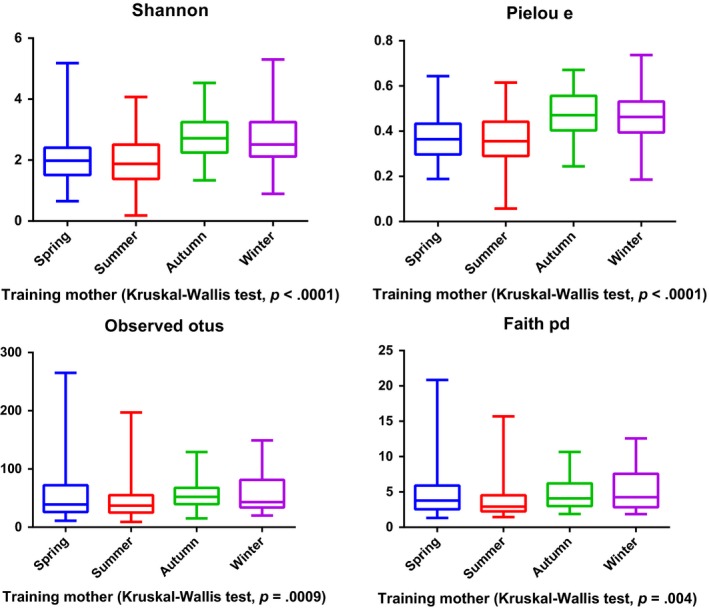
Alpha diversity of gut microbiota in training mother pandas across seasons

**Figure 9:**
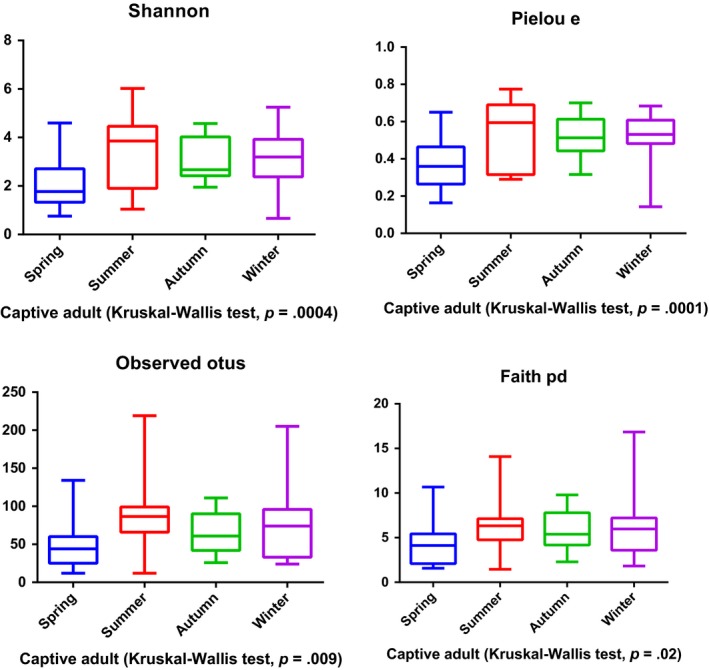
Alpha diversity of gut microbiota in captive adult pandas across seasons

**Figure 10:**
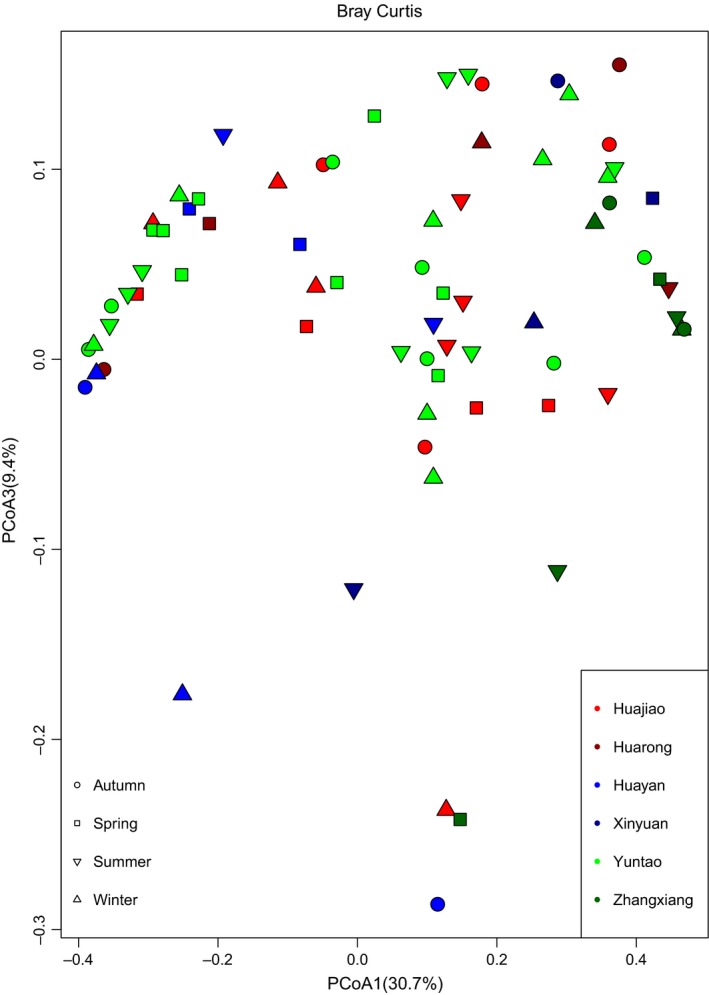
PCoA plot based on Bray–Curtis distance for beta diversity between individuals and seasons. Different colors stand for different individuals. Different shapes stand for different seasons

The intestines of mammals contain millions of various types of bacteria that educate the immune system, digest food, produce vitamins, and promote gastrointestinal (GI) motility (Nicholson et al., 2012). Dysbiosis of the gut microbiota may contribute to immune and neurological disorders, as well as GI problems (Sekirov, Russell, Antunes, & Finlay, 2010). Given the crucial role of gut microorganisms in maintaining GI health, it is necessary to understand the status of the gut microbiota of giant pandas that are undergoing the process of reintroduction. In this study, we found that both the gut microbiota of baby and mother pandas in the training group is similar to that of captive pandas. Also, both the gut microbiota of captive and wild‐training pandas are significantly different from that of wild pandas. Consistent with other studies (Clayton et al., 2016; Kong et al., 2014), our findings reinforced the fact that wild pandas possess the most diverse gut microbiota. After release, the gut microbiota underwent a conversion into that of wild pandas as demonstrated by the panda Zhangxiang. It is reasonable to surmise that when a released panda is exposed to the climate and food of a wild environment such a change is notable. Thus, the environment remains a major factor that influences the development of gut microbiota in pandas. In addition, a previous study reported that the captive panda gut microbiota is highly variable across seasons (Xue et al., 2015), due to their seasonal dietary change in preference for bamboo plant parts (Williams et al., 2013). Our study also supports this conclusion. We found that the alpha diversity of gut microbiota significantly varied across seasons in the training baby group (*p* = .04) (Figure 7), training mother group (*p* < .01) (Figure 8), and captive adult pandas (*p* < .01) (Figure 9). Further, we found the training baby pandas’ gut microbiota stay more similar to themselves than to others across seasons by a PCoA plot (Figure 10) and PERMANOVA test (Table 4). These findings may indicate panda gut microbiota linked with ecosystem stability and individual.

**Table 4:** PERMANOVA for training baby pandas

	Df	Sums of squares	Mean squares	F. Model	Variation (R2)	Pr (>F)
PERMANOVA test						
Individual	5	2.833848	0.56677	3.917806	0.228873	0.001
Residuals	66	9.547891	0.144665		0.771127	
Total	71	12.38174			1	
PERMANOVA test						
Season	3	1.237043	0.412348	2.515962	0.099909	0.011
Residuals	68	11.1447	0.163893		0.900091	
Total	71	12.38174			1	
PERMANOVA test (strata = individual)						
Season	3	1.237043	0.412348	2.515962	0.099909	0.06
Residuals	68	11.1447	0.163893		0.900091	
Total	71	12.38174			1	
PERMANOVA test (strata = season)						
Individual	5	2.833848	0.56677	3.917806	0.228873	0.001
Residuals	66	9.547891	0.144665		0.771127	
Total	71	12.38174			1	

We discovered that the gut microbiota diversity of the wild‐training baby pandas (i.e., the released group) was noticeably different than that of members of the unreleased group. We detected several potentially beneficial bacteria that were more abundant in the released group based on Random Forest and AUC analysis, such as *Roseburia* (Tamanai‐Shacoori et al., 2017), *Coprococcus* (Riviere, Selak, Lantin, Leroy, & De Vuyst, 2016), *Ruminococcus* (Flint, Scott, Duncan, Louis, & Forano, 2012), *Clostridium, Sutterella (*Nguyen et al., 2019 *), Dorea, and Epulopiscium*. Interestingly, most of them are butyrate‐producing bacteria. *Roseburia* is a butyrate‐producing, Gram‐positive, anaerobic bacteria. Suppression of *Roseburia *spp. may affect various metabolic pathways of its host and is associated with several diseases including irritable bowel syndrome, nervous system conditions, and allergies (Tamanai‐Shacoori et al., 2017). *Roseburia *spp. could also serve as probiotics for the restoration of beneficial flora (Tamanai‐Shacoori et al., 2017). In addition, the genus *Coprococcus* belongs to a group of anaerobic cocci that are known to produce butyrate, an essential metabolite in the human colon. Butyrate is the preferred energy source of the colon epithelial cells. It contributes to the maintenance of the intestinal barrier functions and has immunomodulatory and anti‐inflammatory properties (Riviere et al., 2016). *Ruminococcus* is known to degrade and convert complex polysaccharides into a variety of nutrients for their hosts (Flint et al., 2012). Members of the family *Lachnospiraceae* (e.g., f_Lachnospiraceae and g_*Clostridium*) were also regarded as short‐chain fatty acids (SCFA) producers and were more abundant in the gut of released baby pandas. The relative abundance of *Sutterella* was significantly lower in dogs with aggressive behavior than dogs with normal behavior. Between the phobic and aggressive dog group, a slight depletion of the genus *Epulopiscium* was observed in the latter groups (Mondo et al., 2019). Another group noticed that OTUs in the genera *Dorea*, *Ruminococcus*, and *Coprococcus* were significantly more abundant in wild Guizhou snub‐nosed monkeys (*Rhinopithecus brelichi*), in comparison to captive ones (Hale et al., 2019). These potentially beneficial bacteria may be useful as biomarkers to provide evidence for which baby pandas are more suitable for reintroduction, but more samples are needed. Further research is needed to understand the specific role that these beneficial bacteria have in the intestinal tract of released baby pandas.

In summary, we found that the gut microbiota of wild‐training pandas is similar to that of captive pandas and significantly different from that of wild pandas. Also, the gut microbiota of baby pandas gradually becomes more similar to that of wild pandas after being released into the wild. Our results revealed that *Roseburia, Coprococcus, Ruminococcus, Dorea,* and *Sutterella* appeared in high numbers in the babies of successful wild‐training pandas who were released into the wild. These potentially beneficial bacteria may be useful for giant pandas that are more suitable for reintroduction. The gut microbiota may play an important role in panda reintroduction.

## AUTHOR CONTRIBUTIONS

Ying Li designed the research. Jingsi Tang, Chengdong Wang, and Hemin Zhang contributed sample collection and data analysis. Jingsi Tang, Jiangchao Zhao, and Ying Li wrote and revised the paper. Wei Guo, Sudhanshu Mishra, Fanli Kong, Bo Zeng, Ruihong Ning, Desheng Li, Jiandong Yang, Mingyao Yang, Mingwang Zhang, Qingyong Ni, and Yan Li contributed sample processing. All authors read and approved the manuscript.

## Data Availability

The data sets with 16S rRNA gene Illumina HiSeq sequences reported in this study are available with NCBI SRA Accession (SRR9216868‐SRR9217330). The R‐code, Table S1 metadata, and QIIME2 command lines are available at Dryad, https://doi.org/10.5061/dryad.5qfttdz12.
